# Bioadhesive Bacterial Microswimmers for Targeted Drug Delivery in the Urinary and Gastrointestinal Tracts

**DOI:** 10.1002/advs.201700058

**Published:** 2017-05-18

**Authors:** Babak Mostaghaci, Oncay Yasa, Jiang Zhuang, Metin Sitti

**Affiliations:** ^1^ Physical Intelligence DepartmentMax‐Planck Institute for Intelligent Systems70569StuttgartGermany; ^2^ Department of Mechanical EngineeringCarnegie Mellon UniversityPittsburghPA15213USA

**Keywords:** bacteriabots, bioadhesion, drug delivery, microrobots, microswimmers

## Abstract

Bacteria‐driven biohybrid microswimmers (bacteriabots), which integrate motile bacterial cells and functional synthetic cargo parts (e.g., microparticles encapsulating drug), are recently studied for targeted drug delivery. However, adhesion of such bacteriabots to the tissues on the site of a disease (which can increase the drug delivery efficiency) is not studied yet. Here, this paper proposes an approach to attach bacteriabots to certain types of epithelial cells (expressing mannose on the membrane), based on the affinity between lectin molecules on the tip of bacterial type I pili and mannose molecules on the epithelial cells. It is shown that the bacteria can anchor their cargo particles to mannose‐functionalized surfaces and mannose‐expressing cells (ATCC HTB‐9) using the lectin–mannose bond. The attachment mechanism is confirmed by comparing the adhesion of bacteriabots fabricated from bacterial strains with or without type I pili to mannose‐covered surfaces and cells. The proposed bioadhesive motile system can be further improved by expressing more specific adhesion moieties on the membrane of the bacteria.

## 1 Introduction

Over the past decade, there has been an increasing number of studies on cell‐driven biohybrid microswimmers.^1^ Particularly, there is an increasing interest in their potential biomedical applications, including diagnostics and therapy, because of the low production cost and high efficiency, and the capability to program and control such systems.^1^, ^2^ In the case of bacteria‐driven microswimmers (bacteriabots), bacterial cells can be used as the propulsion and sensory units of such biohybrid microsystems. It was shown that the flagellum of the bacteria is a very efficient biomotor,^3^ which in combination with the sensory system of the bacteria, can allow the bacteria to follow variety of environmental stimuli such as chemical, pH, oxygen, and temperature gradients.^3^, ^4^ It was additionally shown that if such bacteria are attached to a synthetic cargo material, it can transport the cargo controlled by its sensory/motor system response.^4^, ^5^

Using bacteria in treating diseases like cancer has a very long history.^6^ It was observed that after intratumoral or systemic injection, the bacteria traced the tumor tissues or cancerous cells, accumulated there, and even colonized the cells, thus leading to regression of the tumors.^7^ Anaerobic bacteria can efficiently find certain types of tumors due to their hypoxic conditions.^8^ However, it was shown that some other types of bacteria can sense the tumor ingredients and precisely accumulate in the proximity of tumor tissue.^9^ Different bacterial species (e.g., *Clostridium novyi*,^10^ *Salmonella* Typhimurium (*S*. Typhimurium),^11^ *Escherichia coli* (*E. coli*),^12^ *Listeria monocytogenes*,^13^ lactic‐acid bacteria family^14^) were investigated regarding their therapeutic effect on different types of diseases such as cancer, diabetes, and colitis. Promising results have led to many human clinical trials, which have shown the effectiveness of this approach.^15^

With respect to above‐mentioned arguments, bacteriabots can be proper candidates for drug delivery. The main concept is attaching bacteria to synthetic functioning parts such as cargo and utilizing bacterial taxis behavior or steering control methods to transport the carried cargos to targeted places. Attachment of different types of bacteria to liposome particles has been investigated. Kojima et al. fabricated the bacteriabots by attaching *Vibrio alginolyticus* to 20 µm liposome particles and showed a significant enhancement in the motility of the particles. They proposed that this system could be further developed to be used as a drug delivery carrier.^16^ Another preliminary study, in microfluidic chemotaxis setups, showed that the bacteriabots, fabricated by attaching *S*. Typhimurium to liposome particles, can have chemo‐ and pH‐taxis behavior and can swim toward the attractants.^17^, ^18^ In a recent ex vivo study, *S*. Typhimurium cells were attached to liposome microparticles encapsulating an antitumor drug, paclitaxel. It was shown that the bacteriabots could sense the cancerous cells or cancerous cell lysates and swim toward them. By attaching the bacteria to liposomes, a significant increase in the average velocity of particles has been observed (from 0.4 to 3.1 µm s^−1^). It was shown that the bacteriabots had higher tumor‐killing efficiency in comparison to bare liposome particles, however, the change in therapeutic output was not extraordinary.^19^ Magnetotactic bacteria were also used to fabricate bacteriabots, which are responsive to both magnetic and hypoxic stimuli. *Magnetococcus marinus* MC‐1 was attached to liposome nanoparticles as potential self‐propelled therapeutic systems. It was observed that around 70 liposome nanoparticles were linked to each bacterial cell. Under the effect of magnetic gradient, a high average speed was recorded for this hybrid system (80 µm s^−1^).^20^ By injecting the microswimmers to the mice bearing a xenograft tumor, it was observed that 55% of the injected bacterial cells (attached to liposome nanoparticles encapsulating drug) penetrated into the hypoxic regions of the tumor by getting help from the magnetic guidance and MC‐1 aerotaxis.^21^

**Figure 1:**
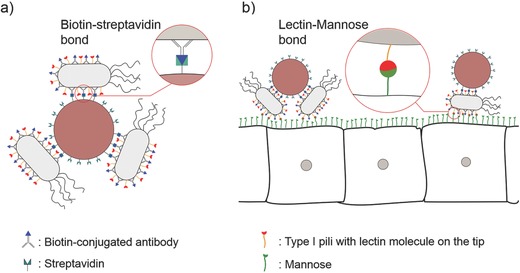
Schematic of bacteriabots a) consisting of synthetic microparticles with attached bacterial cells using the biotin–streptavidin bond and b) attaching to mannose‐expressing disease site cells through lectin–mannose bond. Lectin molecule is located on tip of bacterial type I pili.

For an efficient drug delivery, especially in the case of tumor therapy, one approach can be using tissue anchors to keep the drug‐containing particles close to the site of disease. These anchors could be some antigens or chemicals on the surface of the target tissue cells which can bind to natural or synthetic features on the surface of drug carriers.^22^ In this work, we are using an intrinsic characteristic of *E. coli* bacteria to develop an anchoring system. Most of the wild type *E. coli* strains are covered with type I pili (fimbriae I), which consist of a mannose‐binding lectin group on the tip. Pathogenic *E. coli* bacteria use this lectin group to attach and colonize cells in the case of the urinary tract or some intestinal infections.^23^ This work uses this lectin–mannose bond as a model anchoring system to keep bacteriabots around mannose‐expressing cells. This way, the particles have a longer time to release the encapsulated drugs, which could lead to a more efficient therapeutic effect (**Figure** **1**). So far, there is no report on using bacteriabots as bioadhesive drug carriers. Here in this research, we use microparticles encapsulating a fluorescent dye to show the effectiveness of our approach on attachment of microparticles to a certain type of epithelial cells. This method could be exploited more by investigating more selective attachment moieties and encapsulating therapeutic drugs inside the particles as future works.

There is always a concern regarding the safety of using bacteria or bacteriabots for biomedical applications. This issue has been addressed in many papers, and new methods are constantly being developed to reduce the risk of in vivo applications of bacterial systems.^24^, ^25^ Using attenuated strains which undergo self‐destruction, intratumoral injection instead of systemic injection, and using bacteria in nonsterile organs where the immune reaction is less critical (like intestinal tract), are some of the approaches to use the positive features of bacteria with minimum immunogenicity risk.^26^ In the present work, besides showing the effectiveness of our anchoring system, we aim to investigate the cytotoxicity of the proposed bacteriabots. Moreover, preliminary immunogenicity studies were conducted in order to exploit new ways to reduce such hindering risks.

## 2 Results and Discussion

### 2.1 Characterization of the Fabricated Bacteriabots

The general concept of this study is illustrated in Figure 1. Bacteriabots were fabricated through a biotin–streptavidin bond (Figure 1a). To functionalize the surface of the bacteria, a biotin‐conjugated polyclonal antibody against bacterial lipopolysaccharide (LPS) lipid A was used. Poly(methyl methacrylate) (PMMA) particles with 2.2 µm diameter were functionalized with streptavidin. Figure 1b shows the strategy used to attach bacteriabots to the cells. *E. coli* is covered with type I pili which have lectin molecule on their tip. These lectin molecules have an affinity toward mannose molecules, which are expressed on the surface of certain types of epithelial cells in urinary and intestinal tracts. The microparticles that could potentially contain therapeutic drugs (encapsulating a fluorescent dye in this proof‐of‐concept study) can stay in the proximity of the cells through the attachment of bacteria to the cells. Although, in delivery of drugs, the therapeutic efficiency are influenced by several factors such as encapsulation efficiency and release rate, however, it was reported already that bioadhesive approach could increase the local concentration of drug in the desired location and reduce the side effects.^27^, ^28^ Here, we aimed to show the feasibility of using bacteriabots as bioadhesive drug carriers.

**Figure 2:**
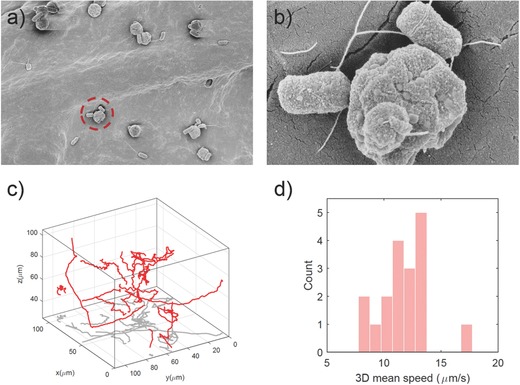
a) Low magnification and b) high magnification SEM images of the bacteriabots. c) 3D swimming trajectories of many bacteriabots recorded by a digital holographic microscope and analyzed by custom particle tracking code (red: 3D trajectories, gray: 2D projection of the 3D trajectories). d) Mean speed distribution analysis based on 3D bacteriabot trajectories.

In the first step, the fabricated bacteriabots were characterized regarding their morphology and motility (**Figure** **2**; Movie S1, Supporting Information). As can be seen in the low magnification scanning electron microscopy (SEM) image in Figure 2a, almost all of the microparticles have bacteria attached to them, which is an indicator of the attachment efficiency. Although we used a conventional biotin–streptavidin protocol to attach bacteria to particles,^29^ to improve attachment efficiency, we used an antibody with a longer spacer arm for biotin conjugation. With this method, we observed a significant increase in the percentage of the particles which were attached to bacteria (Figure S1, Supporting Information). The increased attachment efficiency could be due to the higher accessibility of biotin molecules on the surface of bacteria. Enhancing the attachment efficiency not only increases the mean speed of the microswimmers, but also could potentially increase the biocompatibility of the system as there are less free bacteria and particles after the fabrication, which are not beneficial in therapeutic applications. However, the attachment of bacteria to particles is random, and the bacterial cells are attached to particles with different random numbers (ranging from 1 to 3) and orientations, some from their poles and some from their sides. In the higher magnification SEM image (Figure 2b), it is possible to see that several bacterial cells were attached closely to one particle. In these images, it is possible to see some extent of collapse in the structure of the microparticles. This effect is due to the fact that during the preparation of the SEM samples, we used serial dilution of ethanol, and PMMA particles are not fully resistant to this solvent. Moreover, the particles are hollow, containing fluorescent materials; therefore, shrinkage could be observed in some of the particles.

We analyzed the 3D swimming trajectories of the bacteriabots using a transmission digital holographic microscope (DHM, Figure 2c). DHM records 3D information in a 2D format and reconstructs 3D information numerically afterward. Thus it has high recording speed and memory‐efficiency.^30^ From the obtained trajectories, the mean speed of the bacteriabots was calculated and the mean speed distribution was depicted as shown in Figure 2d. The bacteriabots had an average 3D speed of 11.7 ± 2.2 µm s^−1^. Such speed is higher in comparison to patterned (Janus) and unpatterned *E. coli*‐based bacteriabots (fabricated from particles having the same size) reported in a previous work (9.9 µm s^−1^ for patterned and 7.7 µm s^−1^ for unpatterned bacteriabots, respectively).^31^ One reason could be possibly due to the modified fabrication protocol, which consists of the modification of bacteria with a biotinylated antibody having a longer spacer arm in between which let to better attachment of biotin to streptavidin. The other reason could be collecting the bacteria from the edge of swarm plates, which handed over the fastest bacterial cells.

### 2.2 Attachment of Bacteriabots to Functionalized Surfaces

The attachment mechanism of *E. coli* to the cells through lectin–mannose interaction is well known. The pathogenic bacteria use this mechanism (in combination with other virulence factors) to colonize specific types of epithelial cells in intestinal or urinary tract.^32^ Lectin molecules on the tip of type I pili can bind to the mannose molecules on the surface of the cells, which leads to an anchoring effect.^33^ When bacteria can attach to these types of cells, it is supposed to be possible to keep bacteriabots in the vicinity of the cells through this anchoring competence of the bacteria.

Before investigating the attachment of bacteriabots, we studied the attachment of bacteria to bovine serum albumin (BSA)‐ and mannose‐based functionalized surfaces. The reason we used BSA as a negative control was that the mannose‐functionalization of surfaces was conducted through BSA‐mannose, which is a derivative of BSA. Hence, comparing the attachment of bacteriabots to BSA and BSA–mannose functionalized surfaces can give an impression regarding the effect of mannose functionalization. To show that the attachment is through lectin–mannose binding, a genetically modified strain of the bacteria (*E. coli* ECM1), which does not have any pili on the surface, was used. As it can be seen in Figure S2 (Supporting Information), the number of bacteria (with or without pili) which was attached to the BSA‐functionalized surface and also the number of *E. coli* ECM1 bacteria (without pili) which was attached to the mannose‐functionalized surface are significantly less than the number of pili‐bearing bacteria (*E. coli* MG1655) attached to mannose‐functionalized surface. These drastic differences indicate that the mechanism of attachment between the free bacteria and the mannose‐functionalized surface is through lectin–mannose binding.

**Figure 3:**
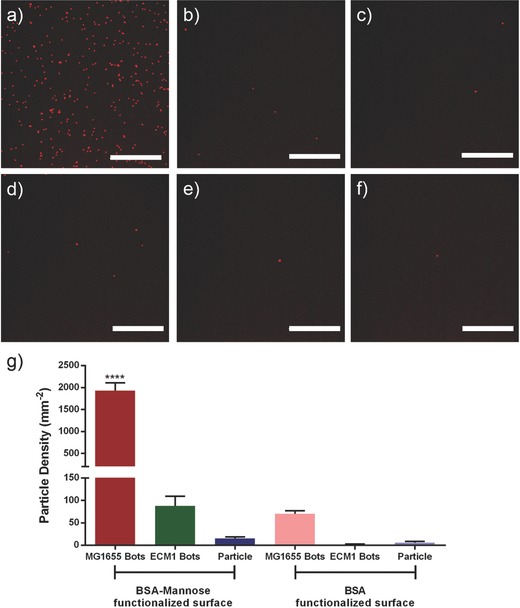
Attachment of particles to different functionalized surfaces: 20× magnification images of a) *E. coli* MG1655‐based (bacteria with pili) bacteriabots, b) *E. coli* ECM1‐based (bacteria without pili) bacteriabots, and c) pristine microparticles attached to mannose‐functionalized surfaces; 20× magnification images of d) *E. coli* MG1655‐based (bacteria with pili) bacteriabots, e) *E. coli* ECM1‐based (bacteria without pili) bacteriabots, and f) pristine microparticles attached to BSA‐functionalized surfaces (scale bar = 20 µm). g) A comparison between the attachment efficiency of different bacteriabots to BSA‐ and mannose‐functionalized surfaces. Kolmogorov–Smirnov test (nonparametric and unpaired *t*‐test) was applied to analyze the results and significant differences (****) were expressed when *p* < 0.05. Error bars indicate standard error of the mean (*n* = 10).

In the next step, the attachment of bacteriabots to the mannose‐functionalized surfaces was investigated. The same negative controls (BSA‐functionalized surface and the bacteriabots which were fabricated from pili‐knock down bacteria) were used (**Figure** **3**). The particle attachment can be observed through the red fluorescent color of the particles. We noticed that the attachment of bacteriabots fabricated from pili‐bearing bacteria (*E. coli* MG1655, Figure 3a) to mannose‐functionalized surface was drastically higher than the attachment of bacteriabots that were fabricated from the bacteria without pili (*E. coli* ECM1, Figure 3b) or pristine particles (Figure 3c). In the case of *E. coli* ECM1‐based bacteriabots or pristine particles, only a few red‐colored particles can be seen on the surface, which indicates the lack of affinity.

To confirm our hypothesis that the adhesion of bacteriabots to mannose‐functionalized surface is through lectin–mannose bond, we checked the attachment of bacteriabots to BSA‐functionalized surfaces. We observed that in the case of *E. coli* MG1655, the number of attached bacteriabots was much lower than the case of mannose‐functionalized surface (Figure 3d). As it was expected, there was not a significant attachment of *E. coli* ECM1‐based bacteriabots (Figure 3e) or pristine particles (Figure 3f) to such surfaces. Figure 3g shows a comparison between the average attachment density of the bacteriabots and particles to mannose‐ and BSA‐functionalized surfaces where the former has over ten times more attachment density than the latter. This plot clearly shows the significant difference in the number of attached bacteriabots as a result of mannose‐functionalization of the surface and presence of pili on the membrane of the bacteria. It can be seen that the attachment of ECM1‐based bacteriabots to mannose‐functionalized surfaces was higher than the attachment of pristine particles to such surfaces. First of all, the number of attached particles in both cases (pristine particles and ECM1‐based bacteriabots) is negligible in comparison to the number of particles attached through *E. coli* MG1655. Also it could be possible that the *E. coli* ECM1 bacteria were still expressing pili in a low extent (or there were few mutated bacteria having pili on the surface). It is worthwhile to mention that all surfaces were washed several times with the flow of phosphate‐buffered saline (PBS), so the presence of the particles is not just a loose precipitation, but it is because of the formation of a bond between bacteriabots and the surface.

A higher magnification image of the bacteriabots attached to a mannose‐functionalized surface can be seen in Figure S3 (Supporting Information). For most of the particles, the attached bacteria can be observed which act as a bridge to anchor microparticles to the surface. In order to show that the attachment of particles to mannose‐functionalized surfaces does not result from precipitation, we conducted dynamic attachment studies (Figure S4, Supporting Information). The mannose functionalization of the PDMS rings was confirmed by staining the rings using fluorescent dye‐conjugated Con A which emitted green color (Figure S4c, Supporting Information). It can be seen in Figure S4d (Supporting Information) that the bacteriabots accumulated on the semivertical mannose‐functionalized wall. Moreover, counting the number of particles attached to the BSA‐functionalized wall and mannose‐functionalized wall shows a different pattern regarding the attachment kinetics. In the case of the BSA‐functionalized surface, the highest density for attached bacteriabots was around 5 × 10^4^ mm^−1^, whereas in the case of mannose‐functionalized surface the bacteriabots density reached above 2 × 10^5^ mm^−1^. There is a fluctuation in the number of attached particles, which could be a result of the detachment of loose bonds over time.

One potential application of the bacteriabots which were fabricated in this study is for oral drug delivery. Different parts of intestinal tract have different pH, from 5.7 to 7.4,^34^ so the bacteria should remain healthy and motile in these pH in order to efficiently propel the bacteriabots. We investigated the health of the *E. coli* bacteria at three different pH by means of different methods. We noticed that the growth of *E. coli* MG1655 slightly decreased when the pH of the environment decreased from pH 7.4 to pH 5.7 (Figure S5, Supporting Information). However, this change in the growth rate was negligible and did not affect the viability and the motility of the bacteria. We analyzed the viability of bacteria using LIVE/DEAD Bacterial Viability Kit at different pH (Figure S6, Supporting Information). According to our results, there is no significant decrease in the viability of the microorganisms when the pH of the environment decreased from pH 7.4 to pH 5.7. Same results were observed with respect to the motility of the bacteria. We analyzed the 2D motility of the bacteria at different pH using an in‐house MATLAB code (Figure S7, Supporting Information). Although the motility slightly decreased by changing the pH of the environment from pH 7.4 to pH 5.7, but this change was not significant.

In order to have functional bioadhesive bacteriabots in the intestine, the lectin–mannose bond should remain stable at different pH. We investigated the efficiency of lectin–mannose bond at different pH by monitoring the interaction of bacteria to mannose‐functionalize surfaces (Figure S8, Supporting Information). According to our investigation, the attachment of the bacteria to mannose‐functionalized surfaces and thus the efficiency of lectin–mannose interaction slightly decreased when the pH of the environment decreases from pH 7.4 to pH 5.7. However, this change was not significant. These results show that the bacteriabots could potentially be bioadhesive in different parts of the intestine.

### 2.3 Attachment of Bacteriabots to Mannose‐Expressing Cells

**Figure 4:**
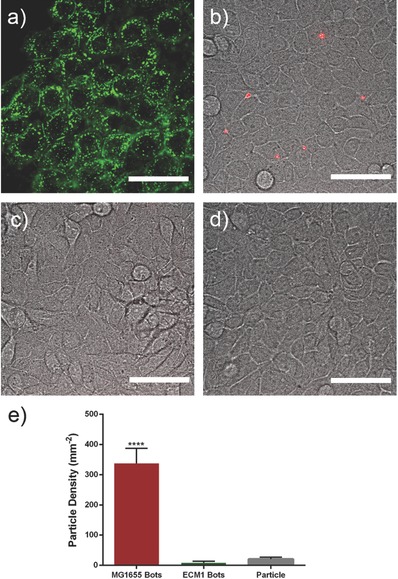
a) Mannose expression on the surface of HTB‐9 cells. The cells were stained using Alexa flour 488‐conjugated Con A. b) Attachment of bacteriabots fabricated from *E. coli* MG1655 (bacteria with pili). c) Attachment of bacteriabots fabricated from *E. coli* ECM1 (bacteria without pili). d) Attachment of pristine particles to HTB‐9 cells (scale bar = 20 µm). e) Diagram comparing the density of the attached bacteriabots to HTB‐9 cells. Kolmogorov–Smirnov test (nonparametric and unpaired t test) was applied to analyze the results and significant differences (****) were expressed when *p* < 0.05. Error bars indicate standard error of the mean (*n* = 10).

Specific eukaryotic cells express mannose on their cell membranes. Pathogenic *E. coli* strains use a lectin–mannose attachment and some other virulence factors to colonize cells in intestinal and urinary tract.^35^ We showed that the bacteriabots that were fabricated from *E. coli* MG1655 (having pili) have the competence to become immobilized on mannose‐functionalized surfaces. It is supposed that this immobilization can be preserved also in the case of mannose‐expressing cells. We used a cell line which was originated from the cancerous urinary bladder tissue and was shown to have mannose expression.^36^ We confirmed the mannose expression by staining the cells with fluorescent dye‐conjugated Con A (**Figure** **4**a). The green color is an indicator of the D‐mannose molecules on the surface of the cells. As a first control step, the attachment of bacteria with pili (*E. coli* MG1655) and the bacteria without pili (*E. coli* ECM1) to the cells was investigated (Figure S9, Supporting Information). It can be seen that there is a significant difference between the numbers of attached bacteria as a result of the presence of pili on the surface of bacteria. In Movie S2 (Supporting Information), it can be observed that in the case of *E. coli* ECM1, there are only a few bacterial cells that are freely swimming over cells. On the other hand, in the case of *E. coli* MG1655, a large number of bacteria attached to cells, which show mostly tumbling movement in their place. It should be considered that, in all cases, the cells were washed three times with Dulbecco's phosphate‐buffered saline (DPBS) in order to get rid of the free (unattached) bacteria or bacteriabots.

To calculate the attachment efficiency, the number of particles encoding a red fluorescent dye (Red4) attached to HTB‐9 cells was counted. Figure 4b shows the attachment of the bacteriabots fabricated from *E. coli* MG1655 to HTB‐9 cells. It can be seen that in this case, several bacteriabots were attached to the cells, whereas the *E. coli* ECM1‐based bacteriabots (Figure 4c) or the pristine particles (Figure 4e) did not have the ability to adhere to the cells. A comparison between the density of attachment (Figure 4f; bacteriabots fabricated from MG1655 strain, bacteriabots fabricated from ECM1, and the pristine particles) can show the significant difference in attachment efficiency. Of course, the density of attached bacteriabots to cells is less than the case of mannose‐functionalized surfaces. It can be seen in Figure 4a that the mannose expression is not homogenous on the surface of the cells and there are regions where there is no (or little) mannose expression and other regions with an accumulation of mannose molecule. On the other hand, on the surface of the cells, there are many molecules and ligands which make the mannose molecules less accessible. Hence, the attachment efficiency is not as high as the case of functionalized synthetic surfaces. As a control, we treated the HTB‐9 cells with Con A, to saturate the surface mannose, and then exposed them to the bacteriabots. As it can be seen in Figure S10 (Supporting Information), there is no particle attached to the surface of treated cells.

With respect to the application of the bioadhesive bacteriabots for drug delivery to the intestinal tract, there are some concerns regarding the viability and motility of the bacteria and controllability of the bacteriabots. Although we showed in section 2.2 that the bacterial cells remain viable and motile at different pH (according to the pH in different parts of the intestine), but in the case of oral administration, the bacteriabots should pass through the stomach containing gastric acid (pH 1.5 to 3.5) and degrading enzymes. Some *E. coli* strains were shown to survive this harsh condition.^37^ In an in vivo study, a probiotic strain of *E. coli* bacteria were administrated to the mice having a cancerous liver. It was observed that the bacteria survive the harsh condition of the stomach and passed through the intestinal barrier to colonize the cancerous liver through its chemotaxis competence.^9^ On the other hand, for oral administration of sensitive therapeutic agents, they are normally encapsulated in degradable protective coatings. Enteric‐coated systems have been extensively studied because they can be tuned to release the drug in a specific part of the intestine after a specific time or by exposing to specific chemicals.^38^ These types of capsules have been already used to deliver probiotic bacteria for therapeutic applications.^39^ Encapsulation of *E. coli* bacteria has also been studied.^40^

The other concern with respect to the intestinal application of bacteriabots is with respect to the presence of mucus layer which makes the motility of the bacteriabots on their way to epithelial cells difficult. It was shown that in addition to affinity toward the epithelial cells, pathogenic *E. coli* bacteria can stick to the mucus layer through lectin–mannose bond at certain pH.^41^, ^42^ This can gives the bacteriabots “mucoadhesive” feature which is a goal in oral drug delivery.^43^ On the other hand, certain strains of *E. coli* are capable to compromise mucus layer and get access to epithelial cells.^44^ Some other bacterial species have enzymes on the surface to locally degrade/disrupt mucus and penetrate into it. Walker et al. used one of this enzyme (from *Helicobacter pylori*) to functionalize a magnetic microrobot in order to give them the competence to penetrate the mucus layer.^45^ In the same way, it is possible to express such enzyme in *E. coli* bacteria (by genetic engineering), and subsequently, fabricate bacteirabots which are able to pass through mucus layer in a short time.

It is worth mentioning that, to have functional medical microrobots, there is a need to implement other features in the bacteriabot system. Although the chemotaxis can guide the bacteriabots toward the site of disease, but it can sense the environment just in a short‐range distance. Some group made hybrid systems by including a magnetic part in the bacteriabots in order to use magnetic actuation/guidance. The magnetic part can be implemented in the synthetic part, or be the natural iron oxide mineralized by the bacteria.^21^, ^46^, ^47^

### 2.4 Cytotoxicity Investigations and Complement Activation Assay

The toxicity generated by the bacteriabots was investigated regarding the cell membrane damage (LDH assay) and changes in the metabolic activity of the cells (MTT assay). Primarily, the toxicity of the individual PMMA particles and the bacteria (with or without pili) toward HTB‐9 cells at a broad range of concentrations was monitored using LDH method (Figure S11, Supporting Information). As expected PMMA as a well‐known biocompatible material did not cause any significant membrane damage even at high concentrations (Figure S7a, Supporting Information). In the case of bacteria, although the cytotoxicity graphs showed a concentration‐dependent pattern, the viability of HTB‐9 cells remained above 80%, in all concentrations of the bacteria, which show the negligible cytotoxicity (Figure S7b,c, Supporting Information). With these promising results regarding the low toxicity of the living and synthetic parts of the bacteriabots, it was expected that the bacteriabots (as a mixture of bacteria and PMMA particles) would also show a nontoxic behavior at the relevant concentrations. Furthermore, we conducted more precise toxicity studies on the bacteriabots, themselves.

**Figure 5:**
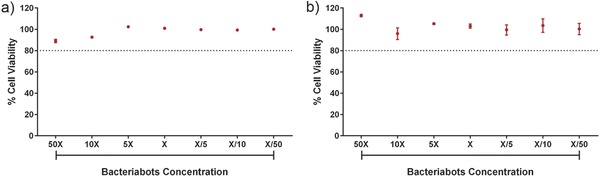
Cytotoxicity of the bacteriabots fabricated from *E. coli* MG1655 bacteria studied by a) LDH and b) MTT assays.

To investigate the toxicity of bacteriabots, the functional concentration (a certain amount of bacteria and particles which was used in attachment studies) and concentrations above and below this value were monitored using LDH and MTT assays (**Figure** **5**). For the concentration that was used for our attachment studies (concentration X), both LDH and MTT showed a full viability of HTB‐9 cells, which indicated the nontoxic behavior of the bacteriabots. For concentrations higher than this value, the viability of the cells was also always above 80%. As a control, we checked the toxicity of the bacteriabots fabricated from the bacteria without pili (Figure S12, Supporting Information), which showed the same results. This work is one of the first studies considering ex vivo characterization of the bacteriabots cytotoxicity. Considering that a full biocompatibility characterization is required before using such systems for biomedical applications, this could be first preliminary results toward dismissing some of the prejudices regarding the toxicity of bacterial systems.

However, a bigger concern of using bacteriabots is not their toxicity, rather their immunogenicity. Injecting bacteria into the parts of the body which are in contact with the immune system (including immune cells) can generate an immune response which could be lethal in severe cases.^15^ The bacteriabot system that we studied in this work is supposed to be hypothetically used in parts of the body where there is much less concern regarding the immunogenicity (the gastrointestinal (GI) and urinary tracts). However, a preliminary study regarding their effect on the activation of complement system was conducted to have a clearer understanding in this regard. C3a complement activation assay, which was conducted according to a standard protocol mentioned in previous studies,^48^ can give some first idea as to what extent the bacteriabots can lead to complement‐activated clearance and trigger immune system. It was already known that the *E. coli* bacteria can induce the complement system,^49^ but the synergic behavior after attaching to microparticles is not known.

**Figure 6:**
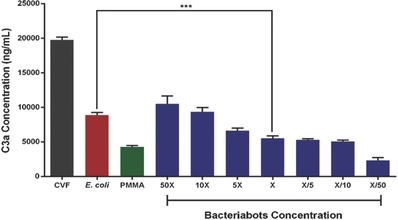
Results of C3a complement activation assay for the bacteriabots at different concentrations. PMMA microparticles and *E. coli* were checked regarding their effects on C3a complement activation at the same numbers used to make the functional concentration of bacteriabots (concentration X).

As can be seen in **Figure** **6**, the bacteriabots in the chosen concentration for the attachment studies (concentration X) partially induced the conversion of C3 to C3a, but this conversion rate was not so much higher than the rate of conversion which was induced by PMMA particles that are made of a recognized biocompatible material. A finding is that the cumulative effect of *E. coli* bacteria and PMMA particles (attached to each other as bacteriabots) on the induction of C3 complement system is significantly less than the effect of the same number of *E. coli* bacteria. There are many factors influencing the immunogenicity of the bacteria. One of the important sources of immunogenicity is LPS of the bacteria^49^ which can induce the activation of complement system. In this work, we covered the LPS of the bacteria using an antibody against LPS, which could be a reason for reducing the immunogenicity. On the other hand, attachment of bacteria to particles can make them less accessible for the proteins involved in triggering of the complement system. This finding can give a new insight regarding the reduction of immunogenicity of bacteriabots. However, more advanced immunological studies are needed for this purpose as a future work.

In this study, we used PMMA as a model drug carrier as the focus was to investigate the bioadhesive feature of the bacteriabots. One important research line in our group is to find novel/smart materials, which are biodegradable and biocompatible, but on the other hand, can be easily attached to bacteria without compromising the motility due to the materials density or surface characteristics. By finding the correct material to fabricate the drug carrier, it might be possible to have triggered‐release of the encapsulated drug, initiated by the bacteria after sensing specific stimuli in future.

## 3 Conclusion

In this work, a new concept for using bioadhesive bacteriabots in targeted drug delivery was proposed and demonstrated. By using lectin molecules, the bacteria not only could attach to mannose‐expressing surfaces and cells, but also in the case of bacteriabots could anchor the particles, which could contain therapeutic drugs. We noticed that the bacteriabots showed an efficient adhesion through the lectin–mannose bond. It was confirmed that this adhesion was not just the effect of precipitation of the particles. Lectin–mannose bond is just one method for anchoring of the bacteriabots, but this anchoring approach can be furthermore exploited by using more precise targeting moieties such as antibodies or even aptamers. Since releasing the drug at the site of disease can significantly increase drug delivery efficiency and suppress parts of side effect, using this active bioadhesive approach could be very helpful for future targeted drug delivery applications of bacteriabots. Moreover, a modified method was used to build bacteriabots. By using the biotin‐conjugated antibody having a longer spacer arm between the antibody and biotin molecule, we increased the efficiency of bacteria‐particle attachment, hence, the bacteriabots had improved motility.

It should be considered that the current bioadhesive system is just a model to show some potential features of the bacteriabots. In order to test the actual “robotic” system, the effect of bioadhesive feature on drug therapeutic efficiency should be examined when the bacteriabot is guided through external stimuli, such as magnetic field or chemotaxis. On the other hand, the bioadhesive feature could be more exploited by expressing certain antibodies on the surface of bacteria, which can be attached specifically to certain antigens.

We started a systematic study on the biocompatibility of bacteriabots. We noticed that the toxicity caused by the bacteriabots in the functional concentrations were negligible. Interestingly, it was shown that fabrication of bacteriabots could even decrease certain aspects of immunogenicity caused by bacteria. Although this topic needs much more investigation, current results could be a primary hint to design bacteriabot systems with reduced immunogenicity by masking their immunogen factors.

## 4 Experimental Section

*Materials and Culture Media*: Antibody against *E. coli* LPS lipid A and the kit for biotin conjugation of the antibody was purchased from Thermofisher (Waltham, USA). In this work, in order to have a higher efficiency of biotin–streptavidin attachment, an NHS‐PEG4‐Biotin having longer spacer arm (29 Å) was used.

The PMMA microparticles that were used to fabricate the bacteriabots were purchased from PolyAn (Berlin, Germany) and have an average diameter of 2.2 µm. The particles were made of poly(methyl methacrylate), functionalized with streptavidin on the surface and contained Red4 fluorescent dye. Motility media in this research was prepared according to literature and consisted 0.01 m potassium phosphate buffer, 0.067 m sodium chloride, and 10^−4^ m ethylenediaminetetraacetic acid (EDTA) (Sigma‐Aldrich, St. Louis, USA).^50^

Detailed information that was used in this study is mentioned in the Supporting Information.

*Bacterial Strains and Cultivation: E. coli* MG1655 which is used to fabricate the bacteriabots was purchased from Coli Genetic Stock Center (Yale University, New Haven, USA). The bacterial strain without pili (ECM1, *E. coli* MG1655Δ*fimA‐H*) was obtained from Prof. Luis Ángel Fernández (Spanish National Center for Biotechnology, Madrid, Spain). The strain, which has a deletion in the operon encoding type 1 pili, is derived from *E. coli* MG1655.^51^

*Fabrication of Bacteriabots*: Bacteriabots were fabricated according to a previous work^29^ with some modifications. The bacteria and particles were washed three times with 1× PBS using centrifugation at 1500 g for 5 min. Antibody against LPS (conjugated with biotin) was added to the bacterial culture to reach a final dilution of 1:50. After 1 h of shaking at room temperature, the bacteria were washed with PBS three times. The particles were washed with PBS three times using centrifugation at 6000 g for 5 min. The bacteria and particles were mixed in motility media and shacked at room temperature for 1 h.

The functional concentration of bacteriabots which was used for attachment studies (concentration X) was optimized after performing a set of attachment and toxicity experiments and prepared by mixing 100 µL bacteria (OD 0.2, 1.5 × 10^8^ cells mL^−1^) and 25 µL particle (0.1% w/v, 3 × 10^8^ particles mL^−1^) in 1 mL motility medium or RPMI 1640 medium.

*Characterization of Bacteriabots*: Detailed information regarding the protocols that were used to characterize the bacteriabots (microscopy, motility analysis, cytotoxicity, and complement activation competence) is mentioned in the Supporting Information.

*Assessment of the Attachment of Bacteriabots to Functionalized Surfaces and the Cells*: For investigating the attachment affinity of bacteria and bacteriabots toward mannose, glass slides were functionalized with BSA and BSA–mannose. For this purpose, the glass surfaces were functionalized with APTES by incubating them with APTES for 45 min at room temperature, followed by rinsing with isopropanol and performing soft‐backing at 120 °C for 5 min.^52^ The APTES‐treated glass slides were incubated with BSA or BSA–mannose solutions (100 µg mL^−1^) for 1 h and rinsed with PBS afterward. The suspensions of the bacteria, particles, and bacteriabots were added to these surfaces and incubated at room temperature for 1 h. The slides were washed using 1× PBS and the density of bacteria and particles attached to the surface was calculated after imaging ten samples using spinning disk confocal microscopy.

Figure S4a (Supporting Information) schematically shows the experimental setup for dynamic attachment studies. The adherence of bacteriabots to BSA‐ or mannose‐functionalized semivertical PDMS surfaces was aimed to monitor. In order to attach BSA or BSA–mannose proteins to PDMS rings, in the first step, the surface should be functionalized with amine groups. For this purpose, PDMS square rings were functionalized with APTES according to a previous study^53^ with some modifications. Briefly, the surface of PDMS was activated using oxygen plasma (Plasma System Zepto, Diener Electronics, Ebhausen, Germany) for 3 min. The PMDS rings were immersed in 10% APTES in absolute ethanol at 50 °C for 2 h. After washing the PDMS rings with water, BSA and mannose functionalization was performed by immersing the rings in 100 µL mL^−1^ solutions of BSA or BSA–mannose in PBS. PDMS rings were washed with PBS in order to get rid of free proteins. The rings were placed on glass coverslips and covered by another coverslip on the top, leaving a hole for adding the bacteriabot solution. Bacteriabots solution was added to the system after placing the system on the confocal microscopy stage. Time‐lapse movie was recorded with 10 s intervals from five samples and the number of fluorescent particles attached to the wall was counted over 2 min period.

Attachment affinity of bacteria and bacteriabots toward a mannose expressing cell line (HTB‐9 cells) was also investigated. First, the mannose expression of HTB‐9 cells was studied by staining the cells using a dye with an affinity toward mannose. For this purpose, the cells were cultivated in a 24 well‐plate at a density of 5 × 10^5^ cells per well. After reaching to 90% confluency, the cells were washed using DPBS. After adding 1 mL solution of Alexa Fluor 488 Conjugated Con A in DBPS (50 µL mL^−1^), the cells were incubated for 15 min at 37 °C. The cells were washed three times using DPBS in order to get rid of free dye and imaged using spinning disk confocal microscopy.

For investigating the attachment of bacteriabots, the cells were seeded in 24 well‐plate at a density of 5 × 10^5^ cells per well. After reaching to 90% confluency, the cells were washed with DPBS. The cells were incubated with bacteria, particles, and bacteriabots (fabricated from *E. coli* MG1655 or ECM1 strains) dispersed in RPMI 1640 medium (without antibiotics) for 1 h, followed by a washing step with DPBS for 3 cycles. The density of bacteria and particles attached to the cells was calculated after performing live‐cell imaging on ten samples using spinning disk confocal microscopy.

As a negative control, HTB‐9 cells were treated with 50 µg mL^−1^ solution of Concanavalin A (Sigma‐Aldrich, USA) for 30 min. After washing the cells for three times with DPBS, the attachment of bacteriabots to the cells was monitored using confocal microscopy.

*Statistical Analysis*: GraphPad Prism software (Ver 6, GraphPad Software Inc., La Jolla, USA) was used for conducting the statistical analysis and plotting the graphs. One‐way ANOVA with Kruskal‐Wallis post‐test was applied to analyze the results. The number of samples and significance criteria is mentioned individually for each test and plot.

## Conflict of Interest

The authors declare no conflict of interest.

## Supporting information

Supplementary

Supplementary

Supplementary
