# Supplementary material for: Bioadhesive Bacterial Microswimmers for Targeted Drug Delivery in the Urinary and Gastrointestinal Tracts
Source: Adv Sci (Weinh). 2017 May 18;4(6):1700058. doi: 10.1002/advs.201700058 (PMC5473323; doi:10.1002/advs.201700058)
Supplement: Supplementary file 1 — Supplementary [file ADVS-4-na-s001.pdf]

Copyright WILEY-VCH Verlag GmbH & Co. KGaA, 69469 Weinheim, Germany, 2017.

ADVANCED SCIENCE
Open Access

Supporting Information

for *Adv. Sci.,* DOI: 10.1002/advs.201700058

Bioadhesive Bacterial Microswimmers for Targeted Drug Delivery in the Urinary and Gastrointestinal Tracts

Babak Mostaghaci, Oncay Yasa, Jiang Zhuang, and Metin Sitti**

DOI: 10.1002/advs.201700058

Copyright WILEY-VCH Verlag GmbH & Co. KGaA, 69469 Weinheim, Germany, 2016.

Supporting Information

Bioadhesive bacterial microswimmers for targeted drug delivery in the urinary and gastrointestinal tracts

Babak Mostaghaci[§,*], Oncay Yasa[§], Jiang Zhuang, and Metin Sitti[*]

[§]Equally contributing first author
[*]Corresponding authors: Babak Mostaghaci (mostaghaci@is.mpg.de), Metin Sitti (sitti@is.mpg.de)

Detailed Materials & Methods

Materials and culture media

LB broth Miller was purchased from Sigma-Aldrich (St. Louis, USA). The swarm plates for cultivating the bacteria contained LB media, 0.45% (w/w) agar (Eiken chemicals, Tokyo, Japan) and 0.5% (w/w) D-glucose (Sigma-Aldrich, St. Louis, USA). Tryptone broth was made of 10 g/L Tryptone (Sigma-Aldrich, St. Louis, USA) and 5 g/L NaCl (Sigma-Aldrich, St. Louis, USA). All media were autoclaved at 121°C for 15 mins. Antibody against *E. coli* LPS lipid A and the kit for biotin conjugation of the antibody was purchased from Thermofisher (Waltham, USA). In this work, in order to have a higher efficiency of biotin-streptavidin attachment, an NHS-PEG4-Biotin having longer spacer arm (29 Å) was used.

The PMMA microparticles, which were used to fabricate the bacteriabots, were purchased from PolyAn (Berlin, Germany) and have an average size of 2.2 μm. The particles were made of poly(methyl methacrylate), functionalized with streptavidin on the surface and contained Red4 fluorescent dye. Motility media in this research was prepared according to

literature and consisted 0.01 M Potassium Phosphate buffer, 0.067 M Sodium Chloride and 10^{-4} M EDTA (Sigma-Aldrich, St. Louis, USA) [32].

For cultivation of HTB-9 cells, an ATCC modified RPMI 1640 was used according to ATCC instruction (Thermo Fischer Scientific, Waltham, USA). The cell culture media was supplemented with 10% (v/v) fetal bovine serum (FBS) and 1% (v/v) penicillin/streptomycin antibiotics (Sigma-Aldrich, St. Louis, USA). The cell toxicity MTT and LDH kits and phosphate buffer saline (PBS and DPBS) were purchased from Thermo Fischer (Waltham, USA). (3-Aminopropyl)triethoxysilane (APTES), bovine serum albumin (BSA) and Concanavalin A (Con A) was purchased from Sigma-Aldrich (St. Louis, USA). A fluorescent version of Con A, which is conjugated with Alexa Flour 488 dye, was purchased from ThermoFischer (Waltham, USA). BSA-Mannose was received from Vector Laboratories (Burlingame, USA).

Bacterial strains and cultivation

E. coli MG1655 which is used to fabricate the bacteriabots was purchased from Coli Genetic Stock Center (Yale University, New Haven, USA). The bacterial strain without pili (ECM1, *E. coli* MG1655Δ*fimA-H*) was obtained from Prof. Luis Ángel Fernández (Spanish National Center for Biotechnology, Madrid, Spain). The strain, which has a deletion in the operon encoding type 1 pili, is derived from *E. coli* MG1655 [33].

The bacterial strains, which are used for the fabrication of bacteriabots, were cultivated on swarm plates to show the highest motility. Both strains were cultured overnight in LB broth at 37 °C, 200 rpm. For *E. coli* MG1655, 25 µL of the bacterial culture was inoculated on the surface of a swarm plate and incubated overnight at 30 °C. The bacteria from the edge of the swarm plate were used to inoculate the tryptone broth which was incubated until it reached to OD_{600} 0.2. For ECM1 strain, due to lack of pili, the bacteria was directly transferred to tryptone broth and incubated at 37 °C until it reached to OD_{600} 0.3.

Growth analysis of the bacteria at different pH conditions

The growth of *E. coli* MG1655 at different pH conditions were investigated using BioTek Synergy 2 Multi-Mode microplate reader. Briefly, single colonies of the bacteria were inoculated into each well of 96-well plate which contained LB media with pH 5.7, pH 6.0 and pH 7.4. Then, the plate was incubated with a slow shaking at 30 °C and absorbance at 600 nm was collected for every 10 min.

Viability analysis of the bacteria at different pH conditions

The viability of *E. coli* MG1655 at different pH conditions was investigated using Molecular Probes *LIVE/DEAD*® *BacLight*™ bacterial viability kit. The experimental was performed as provided by the company with slight changes. Briefly, *E. coli* MG1655 was cultured overnight in different LB media with pH 5.7, pH 6.0 and pH 7.4 at 30 °C, 200 rpm. 5 mL of the bacterial cultures were concentrated by centrifugation at 10000 x g for 10 min. After that, the pellets were resuspended in 1 mL of 0.85% (w/v) NaCl, and 500 μL of these suspensions were added into new tubes which contained 10 mL of 0.85% (w/v) NaCl. The samples were incubated for 1h at room temperature by mixing in every 15 min. At the end of the incubation period, the samples were collected by centrifugation at 10000 x g for 10 min, and then washed 2 times with 0.85% (w/v) NaCl. 1 mL of each bacterial suspensions were mixed and incubated with 3 μL of the dye, which was prepared by combining equal volume of Component A and Component B, at room temperature for 15 min. Finally, 5 μL of the stained bacterial suspensions were investigated with Nikon Eclipse Ti-E spinning disk confocal microscope using 60x oil immersion objective.

Motility analysis of the bacteria at different pH conditions

The motility of *E. coli* MG1655 at different pH conditions was analyzed using an in-house MATLAB tracking code. Briefly, 25 μL of the bacterial culture was inoculated on the surface of a swarm plate and incubated overnight at 30 °C. Then, 25 μL of the bacteria from the edge of the swarm plate was inoculated into different LB media with pH 5.7, pH 6.0 and pH 7.4, and incubated until they reached to OD_{600} 0.2. After that, the bacterial cultures were

10 times diluted in their respective motility buffers which had different pH conditions (pH 5.7, pH 6.0 and pH 7.4). Finally, the bacterial motility was checked under Leica DMi8 inverted microscope in micro-chambers using 40x water immersion objective and the videos, used for the analysis, were recorded at 50 fps.

Interaction of the bacteria with BSA-mannose coated surfaces at different pH conditions

The attachment of the bacteria to BSA-mannose functionalized surfaces at different pH conditions was performed as described before. Briefly, the glass surfaces were functionalized with APTES by incubating them with APTES for 45 min at room temperature, followed by rinsing with isopropanol and performing soft-backing at 120 °C for 5 min. The APTES-treated glass slides were incubated with BSA-mannose solutions (100 µg/mL) for 1 h and rinsed three times with PBS afterward. The suspensions of the bacteria were added to these surfaces and incubated at room temperature for 1 h. The slides were washed three times using 1X PBS and the density of bacteria attached to the surface was calculated after imaging 12 samples using spinning disk confocal microscope.

Toxicity analysis of the bacteriabots

The cytotoxicity of bacteriabots was analyzed using MTT and LDH assays over a broad range of concentration of bacteria, particles, and bacteriabots. For this purpose, the cells were seeded in 96 well-plate at a density of 10^4 cells/well and incubated to reach to 90% confluency. The cells were washed using DPBS buffer. Different concentrations of bacteria, particles, and bacteriabots suspended in RPMI 1640 media were added to the cells and incubated for 4 h at 37 °C. Pure RPMI 1640 was used as positive control and 1% Triton X-100 was added as a negative control. After 4 h, the supernatant was separated and incubated with LDH reagent for 5 min, followed by reading the absorbance at 492 nm using microplate reader (Synergy2, Biotek, Winooski, USA). The cells were incubated with MTT reagent for another 4 h, followed by dissolving cells using DMSO to release the formazan content. The

absorbance was read at 550 nm using a plate reader. The absorbance of positive and negative controls was considered as 100% and 0% viability of the cells.

Assessment of the immunogenicity of the bacteriabots

To investigate the effect of the bacteriabots on activation of the complement system, a C3a complement ELISA kit (Quidel, San Diego, USA) was used according to the manufacturer instructions. For this purpose, 100 µL aliquots of normal human serum (NHS, Quidel, San Diego, USA) were incubated with different concentrations of bacteria (with or without pili) and bacteriabots for 30 min at 37 °C. 10 µL of EDTA (220 mM) was added to NHS as a positive control, and 3 µL of cobra venom factor (CVF, Quidel, San Diego, USA) was added to NHS as a negative control. CVF is supposed to activate the whole C3a content. 100 µL of 5000 times diluted samples were added to the 96-well ELISA plate (containing a murine antibody) and the plate was incubated for 1 h at room temperature. The wells were washed 4 times before100 µL of conjugate solution (contains horseradish peroxidase-conjugated polyclonal antibody to C3a) was added to each well. The plate was incubated at room temperature for 1 h and washed afterward. 100 µL of substrate solution (containing 3,3′,5,5′-tetramethylbenzidine and H_2O_2) was added to each well. After incubating the plate at room temperature for 15 min (protected from light), 100-µL stop solution (1 N HCl) was added to each well. The C3a concentration was calculated after reading the absorption of each well at 450 nm using a microplate reader. The results were normalized considering the positive control as 0 ng/mL C3a.

Supplementary Figures

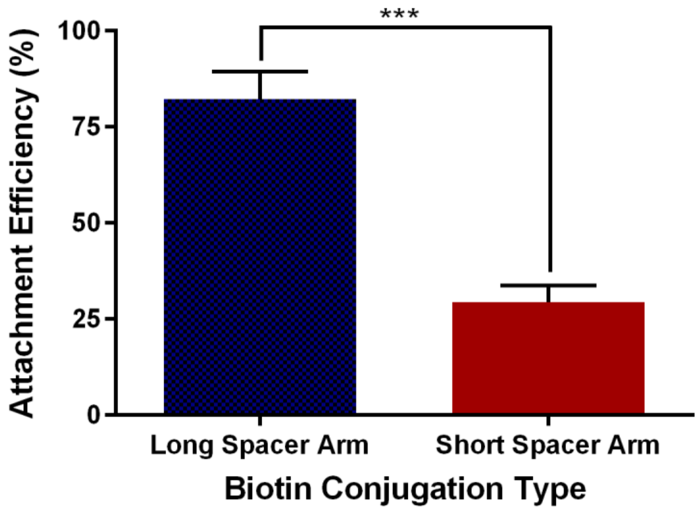

Figure S1. The effect of biotin-conjugation type (using long or short spacer arms) on the attachment efficiency of bacteria to particles. Kolmogorov-Smirnov test (nonparametric and unpaired t test) was applied to analyze the results and significant differences (***) were expressed when $p<0.05$. Error bars indicate standard error of the mean (SEM, n=6).

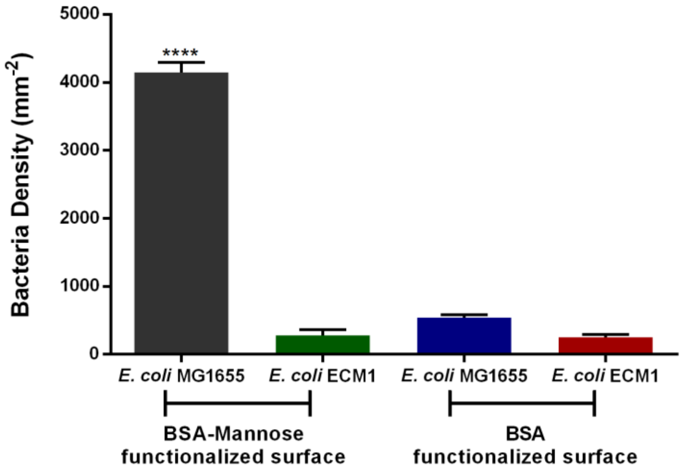

Figure S2. The density of bacteria with or without pili (*E. coli* MG1655 and *E. coli* ECM1) attached to different surfaces, functionalized with BSA and BSA-Mannose. Kolmogorov-Smirnov test (nonparametric and unpaired t test) was applied to analyze the results and significant differences (****) were expressed when $p<0.05$. Error bars indicate standard error of the mean (SEM, n=10).

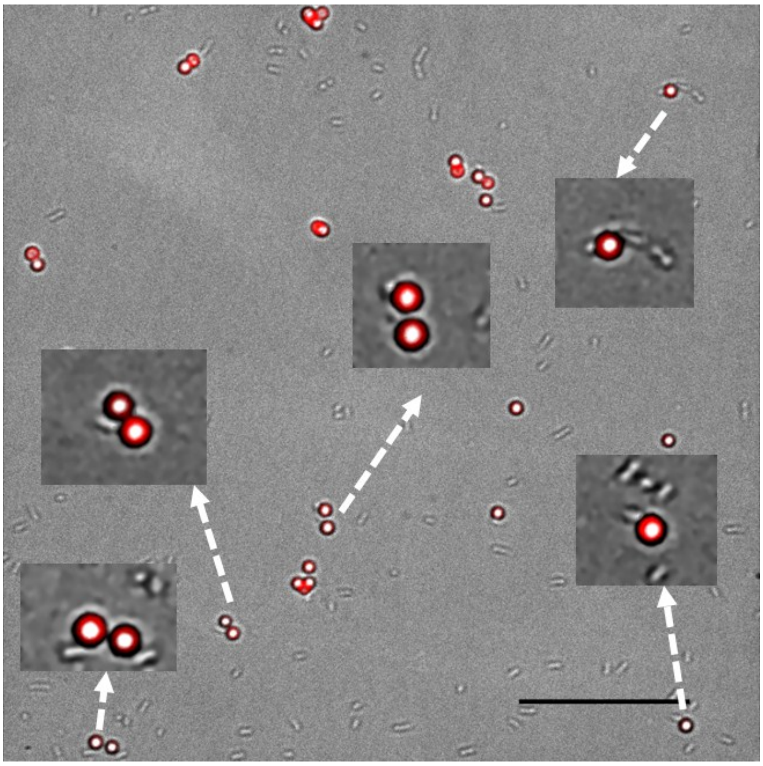

Figure S3. Microscopy images of the bacteriabots attached to mannose-functionalized surface; the inlays show higher magnification images of the particles, which are anchored to the surface through the attached bacteria (scale bar: 20 µm).

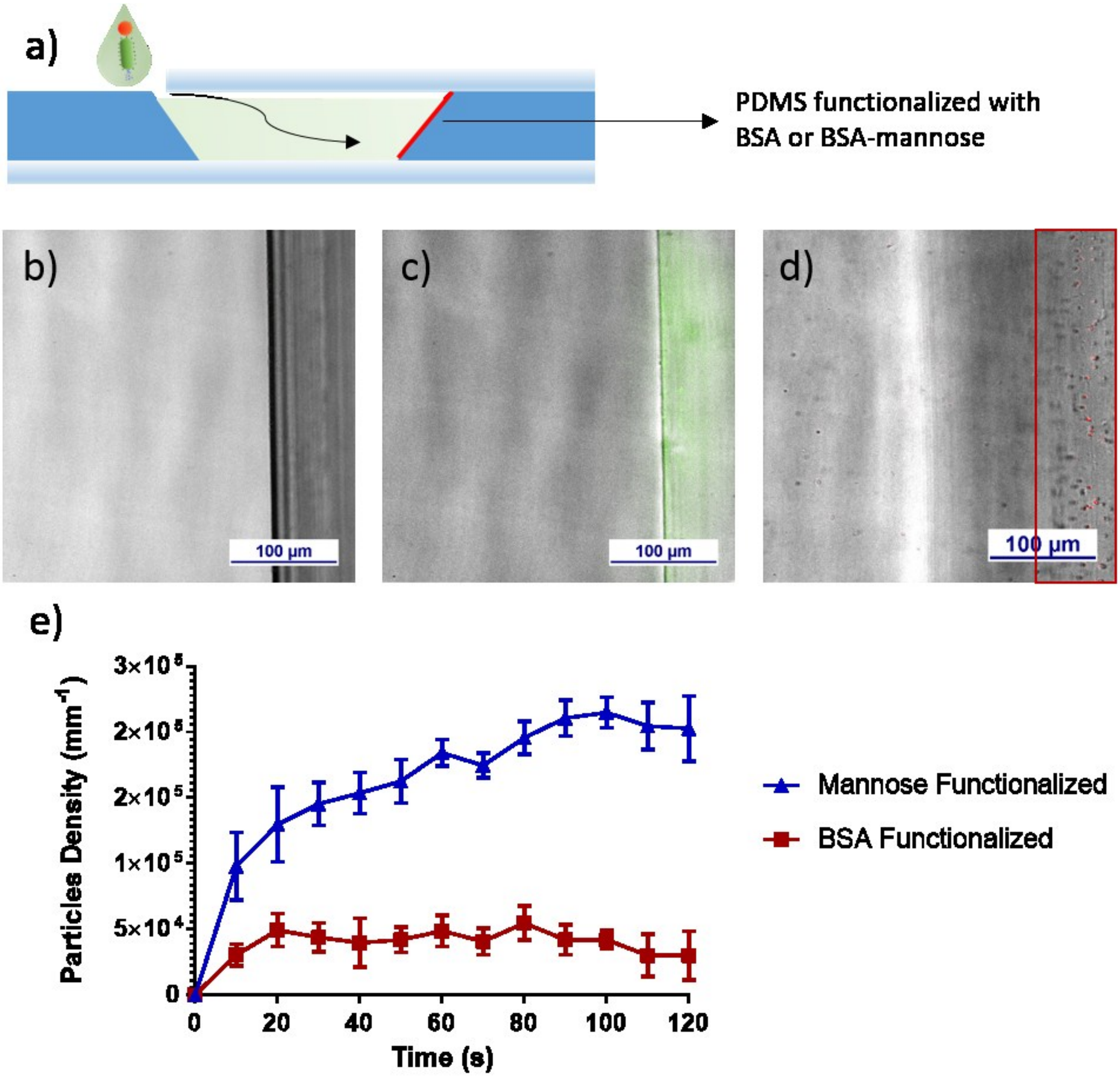

Figure S4. (a) Schematic design of the setup used for dynamic attachment experiment; the PDMS rings were functionalized with (b) BSA and (c) BSA-Mannose and the functionalization was confirmed by staining the rings using Con A-conjugated Alexa Fluor 488; (d) attachment of bacteria and bacteriabots (E to the PDMS ring. The red dots are fluorescent particles which were used to fabricate bacteriabots; (e) attachment of bacteria to BSA- and mannose-functionalized semi-vertical PDMS surfaces over time; error bars indicate standard error of the mean (SEM, n=5).

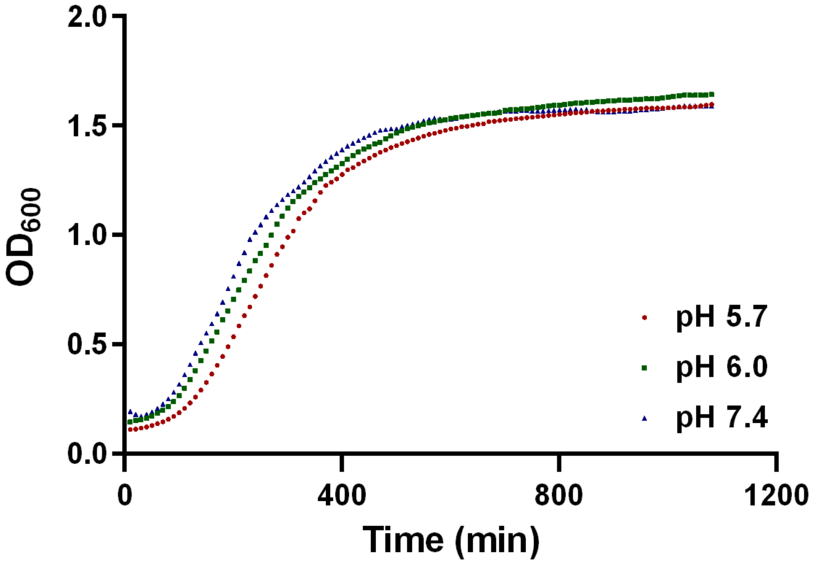

Figure S5. Growth curves of *E. coli* MG1655 cultured overnight in LB media with different pH conditions. Each data point was obtained every 10 min (n=4).

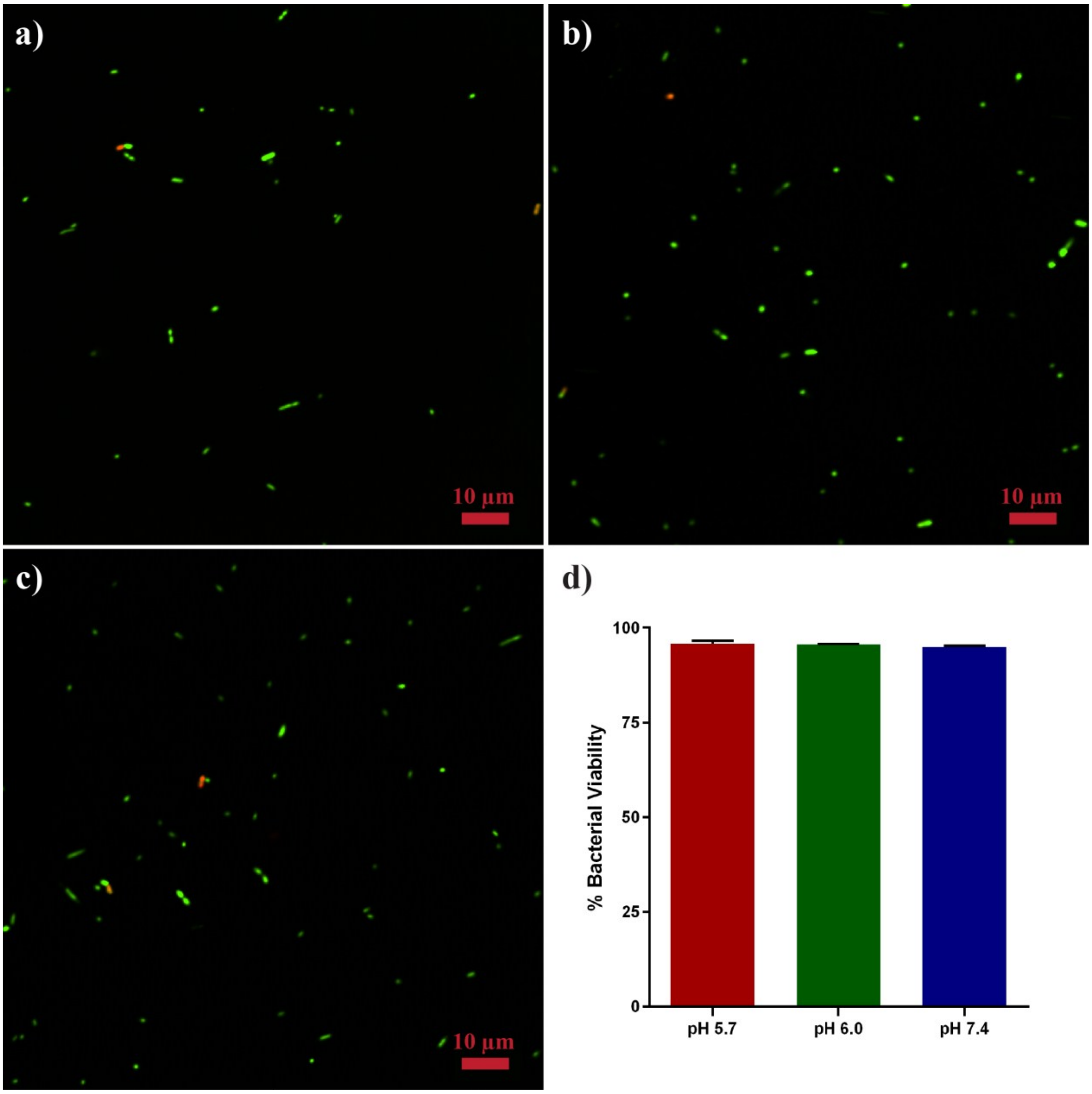

Figure S6. The viability of *E. coli* MG1655 cultured overnight in LB media at different pH. Live/dead microscopy images of the bacteria when cultured at (a) pH 5.7, (b) pH 6.0, and (c) pH 7.4. (d) The bacterial live to dead ratio after incubating at different pH (n=4).

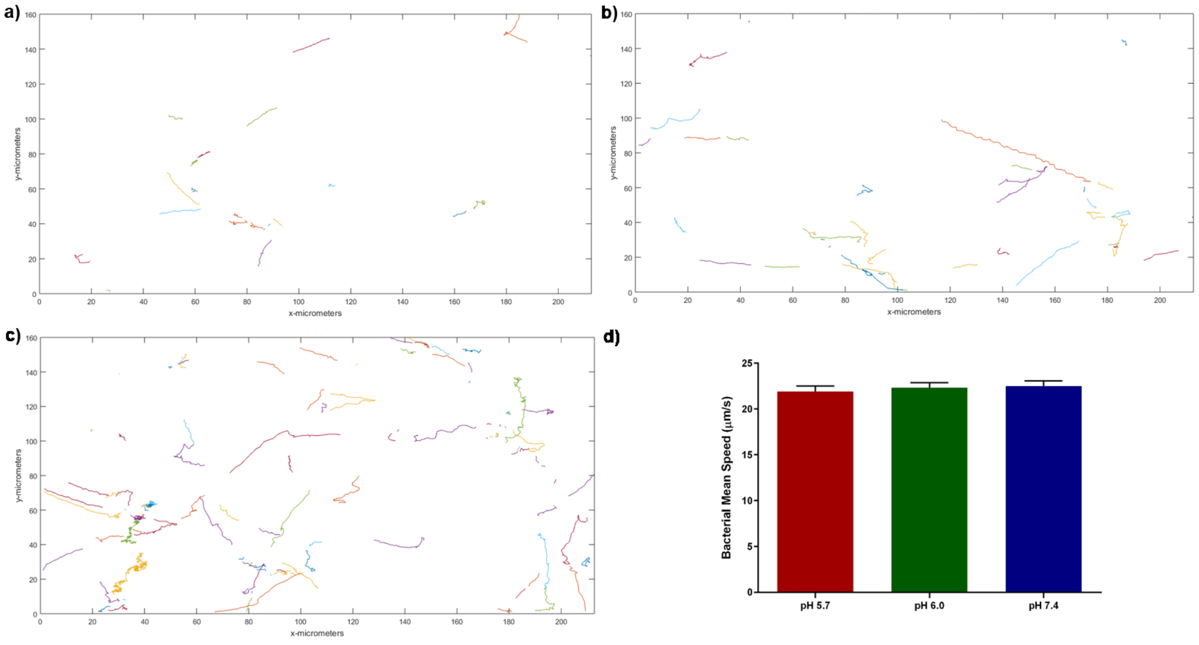

Figure S7. Motility analysis of *E. coli* MG1655 at different pH. 2D trajectory images of bacterial motion after incubating in motility mediums at different pH: (a) pH 5.7, (b) pH 6.0, and (c) pH 7.4. (d) The bacterial swimming mean speed at different pH.

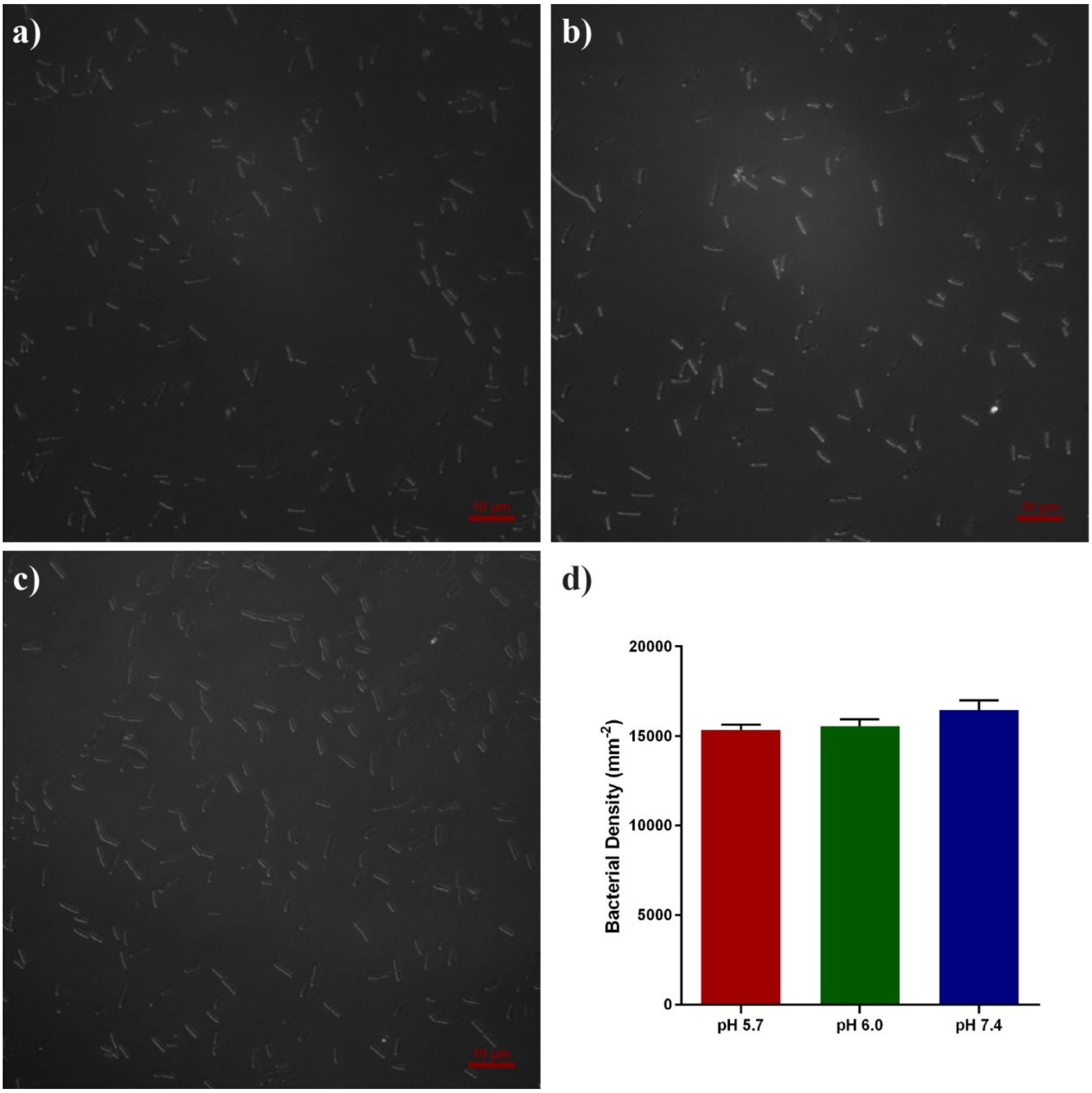

Figure S8. Interaction of *E. coli* MG1655 with BSA-Mannose functionalized surfaces at different pH. Immobilization of bacteria on the surface after incubating them on the mannose-functionalized surface at different pH: (a) pH 5.7, (b) pH 6.0, and (c) pH 7.4. (d) The bacterial attachment density on BSA-Mannose functionalized surfaces when they were cultured and incubated at different pH (n=4).

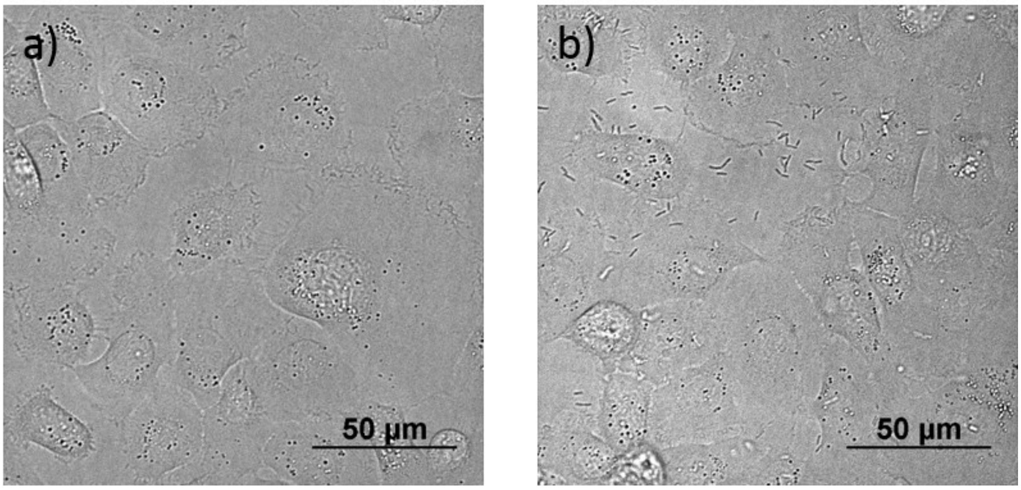

Figure S9. Attachment of (a) *E. coli* ECM1 and (b) *E. coli* MG1655 to HTB-9 cells.

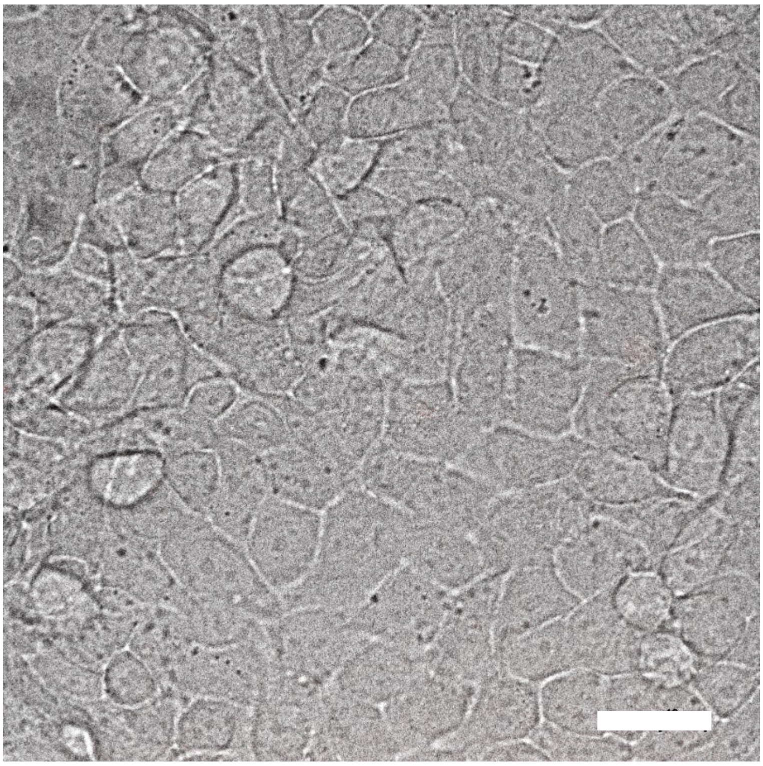

Figure S10. HTB-9 cells treated with Concanavalin A and exposed to MG1655-based bacteriabots. It can be seen that no particles attached to the cells (scale bar=20 μm).

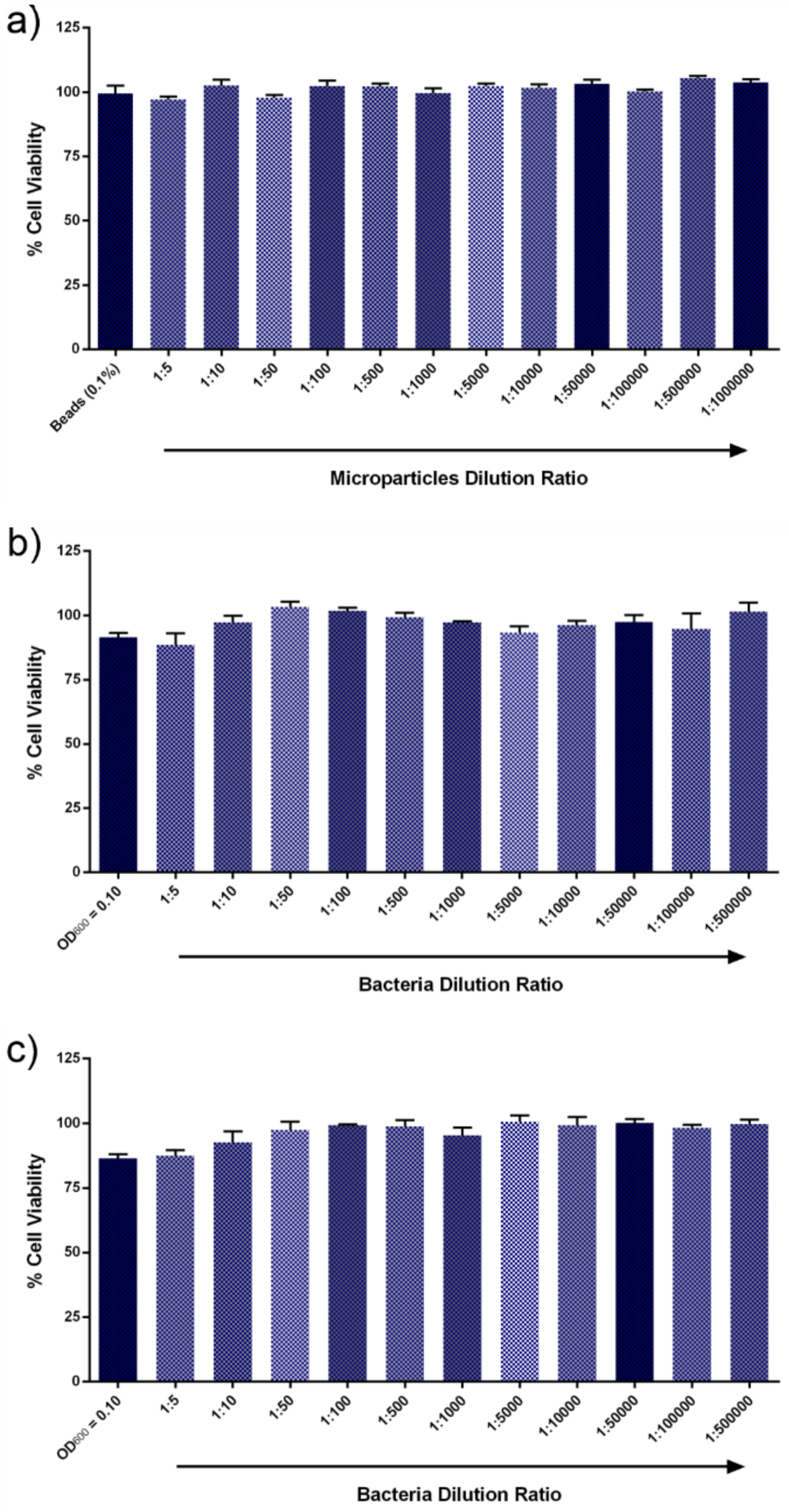

Figure S11. Cytotoxicity of different concentrations of (a) 2.2 μm PMMA particles, (b) *E. coli* MG1655, and (c) *E. coli* ECM1 toward HTB-9 cells analyzed by LDH assay. LDH activity was monitored after 4 h incubation of the cells with the samples (n=3, 0.1% Bead=3×10^8 particles/mL).

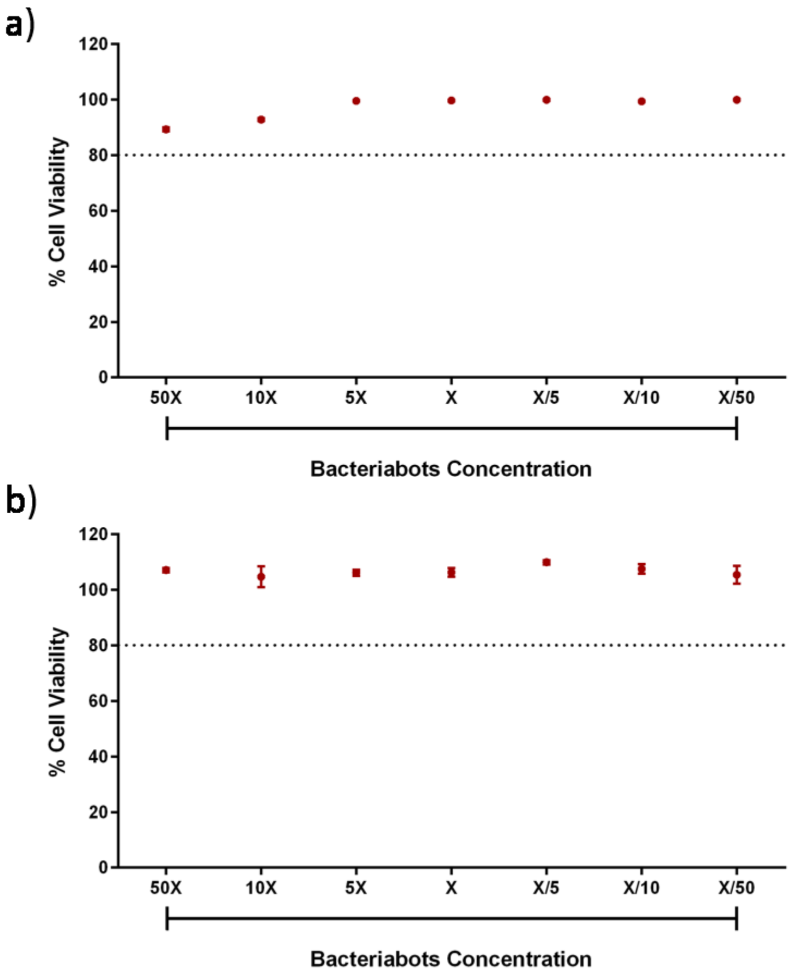

Figure S12. Cytotoxicity of bacteriabots, which were fabricated from 2.2 µm PMMA particles and *E. coli* ECM1 toward HTB-9 cells, analyzed by (a) LDH and (b) MTT methods. Concentration X is the optimum concentration, which was used in the attachment experiments according to materials and method part.

Supplementary Movies

Movie S1. Phase contrast and fluorescent movies taken from bacteriabots fabricated from 2.2 μm PMMA particles and *E. coli* bacteria, which attached to each other using biotin-streptavidin bond.

Movie S2. Attachment of E. coli ECM1 (without pili) and E. coli MG1655 (with pili) to HTB-9 cells.
